# Rethinking positional nystagmus: beyond BPPV

**DOI:** 10.1007/s00415-025-13335-2

**Published:** 2025-09-05

**Authors:** Amanda J. Male, George Korres, Nehzat Koohi, Diego Kaski

**Affiliations:** 1 SENSE Research Unit, Department of Clinical and Movements Neurosciences, UCL Institute of Neurology, 33 Queen Square, London, WC1N 3BG UK; 2 Department of ENT/Neuro-Otology, Attikon University Hospital, Athens, Greece

**Keywords:** Positional dizziness, Positional nystagmus, BPPV, Central positional nystagmus

## Abstract

Positional nystagmus is a frequently encountered yet often underappreciated clinical sign that provides critical insights into vestibular and central nervous system function. For the general neurologist, recognising and correctly interpreting positional nystagmus can significantly impact diagnostic accuracy and guide appropriate management of common and complex dizziness presentations. The current diagnostic framework for positional nystagmus disproportionately favours BPPV, underestimates central positional nystagmus (CPN), and over-relies on imaging. We argue for a paradigm shift that acknowledges clinical complexity, defines objective diagnostic thresholds, and strengthens neurological training.

## Introduction

Dizziness is among the most frequent symptoms encountered by neurologists, with positional dizziness accounting for nearly 50% of dizziness-related consultations in clinical practice [1]. The majority of these cases are attributed to benign paroxysmal positional vertigo (BPPV), a condition with a well-characterised peripheral mechanism involving dislodged otoconia within the semicircular canals. BPPV typically responds well to canalith repositioning manoeuvres such as the Epley or Semont [2, 3], reinforcing its dominance in both clinical focus and published literature. However, BPPV is not the sole cause of positional dizziness.

Central positional nystagmus (CPN) is nystagmus arising due to pathology affecting the central vestibulo-cerebellar pathways. The nystagmus is triggered by and after a change in head position in respect to gravity and is a clinical sign that can closely mimic BPPV [4, 5]. However, because CPN results from fundamentally different pathophysiology it therefore demands a distinct diagnostic approach.

CPN is a common clinical sign observed across a range of neurological disorders, including diffuse cerebellar disease (∼15%), vestibular migraine during attacks (up to 90%), acute central vestibular syndrome (∼33%), isolated cerebellar stroke (∼50%), infratentorial tumours (20–78%), and multiple sclerosis (∼6%) [see 6 for references]. Given its frequency and broad association with central nervous system pathology—including potentially life-threatening conditions such as cerebellar stroke and brain tumours—neurologists must be vigilant in recognising CPN. Awareness of this sign is essential not only for distinguishing central from peripheral causes of vertigo but also for ensuring timely diagnosis and management of serious underlying conditions that may otherwise be overlooked.

### Pathophysiology and aetiology of CPN

In the recent years, mathematical models and detailed neuroimaging have provided further insights into the underlying mechanisms of CPN [6–8]. It is thought to result from abnormal central integration of otolith and semicircular canal inputs, possibly involving dysfunction in velocity storage mechanisms [9, 10]. Lesions affecting the cerebellar nodulus, uvula, or vestibular nuclei are common culprits [8, 11]. Aetiologies of CPN span a spectrum from acutely sinister “structural” causes—such as stroke, demyelination, and posterior fossa tumours—to “nonstructural,” chronic, and often more subtle syndromes, including vestibular migraine [4, 6, 8].

### Clinical presentation and pitfalls

Although CPN may account for up to 12% of patients with positional nystagmus [12], it remains under-recognised in general neurological practice. One reason is that CPN is often a diagnosis of exclusion, where features of the nystagmus do not fit with BPPV. There are no universally accepted diagnostic criteria and there is increasing awareness that nystagmus characteristics alone can be insufficient in distinguishing CPN from peripheral positional nystagmus. This contributes to diagnostic uncertainty and delays in appropriate care.

Patients with CPN may exhibit central ocular motor abnormalities outside of positional nystagmus, including gaze-evoked nystagmus, broken smooth pursuit, and hypermetric saccades [6, 8, 12]. These signs, however, may only become evident with visual fixation removed (e.g., using Frenzel goggles)—a step not routinely performed in most clinical settings. As a result, clinicians may misdiagnose CPN as refractory or atypical BPPV if subtle central ocular motor signs have not been picked up.

Importantly, atypical variants of BPPV (e.g. anterior canal, cupulolithiasis, or apogeotropic forms) can mimic the nystagmus patterns seen in CPN [13, 14]. Without a high index of suspicion for a central disorder and specialised assessment skills, distinguishing between them is difficult. Neurologists in particular should exercise a high level of suspicion for CPN in patients with positional vertigo who have not responded to positional manoeuvres or have atypical features (positional nystagmus without vertigo, associated central ocular motor or other neurological signs, vomiting, or vascular risk factors [8, 15, 16].

### Structured assessment

**Fig. 1:**
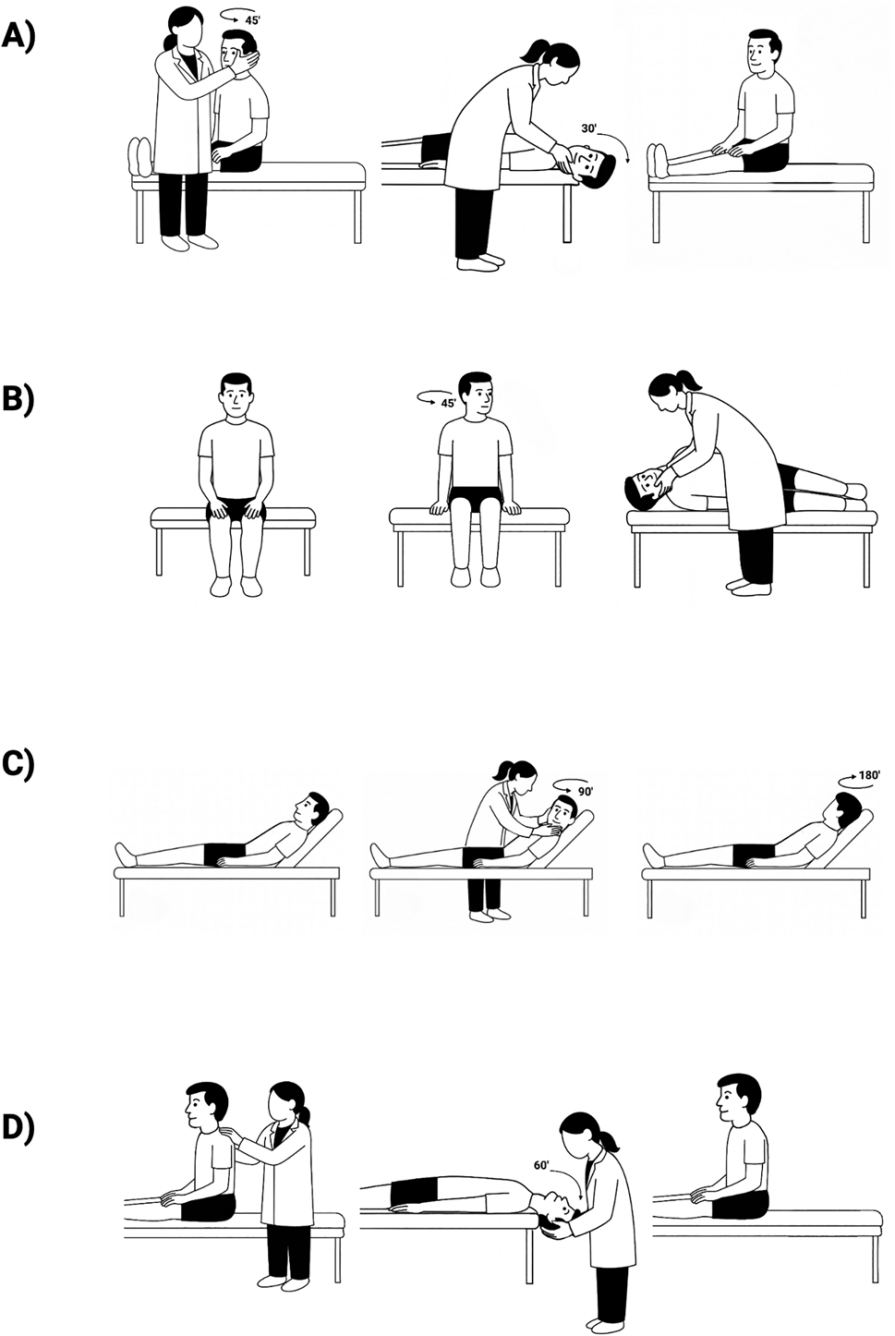
Positional tests used to differentiate BPPV and CPN. **A** Dix–Hallpike test for left ear. Head is turned 45⁰ to the left and then the patient lays down onto their back on the bed, with the neck positioned into 30⁰ extension to observe for nystagmus. **B** Side lying test for left ear. Head is turned 45⁰ to the right and the patient lays down onto their left shoulder into extension of the neck to observe for nystagmus. **C** Roll test for left and right ear. The patient lays on their back with the head of the bed elevated 30⁰. Head is turned 90⁰ to the left and then 90⁰ to the right to observe for nystagmus. **D** Straight head-hanging test. The patient lays down with head straight and the neck is positioned into 60⁰ extension over the end of the bed to observe for nystagmus. (Figure created by EBT Assis, 2025)

Differentiating BPPV from CPN requires a methodical and comprehensive vestibular assessment. Clinicians should assess all six semicircular canals using the appropriate positional tests (see Fig. 1):

- Posterior canal: Dix–Hallpike [17] or side-lying test [18.]
- Horizontal canals: Supine roll test [19.]
- Anterior canals: Straight head-hanging test [20].

These manoeuvres align each canal with gravity, triggering endolymphatic flow that can provoke positional nystagmus in the presence of dislodged otoconia. However, interpretation depends on the clinician’s ability to recognise the significance of the elicited eye movements.

Positional nystagmus represents a diagnostic dichotomy that obscures its true complexity. Clinicians frequently default to a binary diagnostic model: “typical” nystagmus patterns imply a peripheral origin, while “atypical” ones are either dismissed or misclassified as atypical variants of BPPV. This approach risks missing central causes that may closely resemble peripheral variants in clinical presentation.

Atypical BPPV variants—including apogeotropic horizontal canal BPPV, anterior canal BPPV, and cupulolithiasis—are increasingly reported [2, 3, 13]. These forms can closely mimic CPN in both pattern and behaviour [14], making accurate interpretation more difficult for clinicians without advanced training in ocular motor assessment. As a rule of thumb, we would recommend assuming a central cause for such atypical variants. Where neuroimaging is normal and there are no other central signs on examination or in the history, an atypical variant can be considered but here perhaps one should ensure close follow-up and monitoring. Where possible, obtain appropriate consent and record the positional nystagmus using a smartphone (ideally with assistance from a colleague), to facilitate expert review and for documentation.

### Diagnostic challenges in practice

Accurate interpretation of positional nystagmus requires knowledge of neuroanatomical correlates, nystagmus vector analysis, and vestibular physiology—skills often underrepresented in standard neurology or medical training [15]. As a result, clinicians with limited exposure to vestibular disorders may struggle to identify the positional nystagmus characteristics of central pathology and also other central ocular motor signs.

Unlike BPPV, the nystagmus seen in CPN typically lacks latency and persists for longer while the head is maintained in the triggering position [8, 15]. These features are easily misinterpreted as cupulolithiasis variants of BPPV. Directional characteristics can also be diagnostically informative; for example, if the nystagmus direction is inconsistent with the stimulated canal, a central origin should be considered [6]. Moreover, direction-changing nystagmus (a change in the direction of the nystagmus even when the head is still in the same position) cannot occur with BPPV where the direction of the nystagmus is driven by the gravity-dependent cupula displacement. Still, controversy remains, and no single set of features has achieved consensus as a reliable CPN diagnostic signature.

Clinical features associated with CPN that point to a central cause include positional vomiting and a lack of response to repositioning manoeuvres [6, 8]. However, the absence of standardised thresholds for defining “treatment failure” in BPPV complicates matters. Is one unsuccessful repositioning manoeuvre enough to question a BPPV diagnosis, or should multiple trials be attempted? These ambiguities hinder diagnostic clarity, particularly when clinical documentation is incomplete or fragmented across care settings. There is no consensus on this currently, but we believe this is an important area that future research should address.

### The imaging fallacy

Another common misconception is that normal neuroimaging excludes central causes. Although CPN is often associated with identifiable lesions in the cerebellum or brainstem, vestibular migraine—one of the most common central causes—typically yields normal MRI findings. Yet, it accounts for positional nystagmus in up to 90% of symptomatic episodes [4, 6]. This underscores the importance of a thorough history and clinical examination to identify features suggestive of vestibular migraine accompanying CPN.

Whilst the mechanisms responsible for positional nystagmus in vestibular migraine are unknown, theories suggest functional vestibular network disturbances, possibly due to cortical spreading depression, brainstem disinhibition, or impaired otolithic integration of gravity signals [6]. We propose that in disorders such as vestibular migraine, dynamic, nonstructural mechanisms such as ion channel dysfunction (channelopathies) and altered network excitability may be potential contributors to CPN but further research is needed to explore such mechanisms further. Regardless, over-reliance on imaging not only risks false reassurance but may delay appropriate diagnosis and therapy.

### Recommendations

To improve diagnostic accuracy and reduce missed or delayed diagnoses of CPN, we propose four key clinical reforms:

1. Define evidence-based thresholds for treatment failure in BPPV, to avoid premature conclusions in cases of nonresponse.
2. Recognise CPN as a distinct clinical sign, not merely a diagnosis of exclusion, and develop criteria that promote proactive interpretation.
3. Standardise eye movement assessments, including testing with and without fixation, ideally incorporating video-oculography where available.
4. Integrate vestibular and ocular motor assessment training into neurology and general medical education to build clinician confidence and competence.

## Conclusion

The long-standing assumption that positional nystagmus always indicates BPPV is increasingly insufficient. Central positional nystagmus is not rare—it is simply under-recognised. As an important indicator of cerebellar or brainstem dysfunction, CPN must be approached with structured clinical reasoning and vigilance. Moving beyond a binary diagnostic framework will require sharper observation, better training, and a healthy scepticism of “normal” imaging in the face of atypical clinical signs.

## Data Availability

Not applicable.
